# Complex functional brain network properties in anorexia nervosa

**DOI:** 10.1186/s40337-022-00534-9

**Published:** 2022-02-05

**Authors:** Arpana Gupta, Ravi R. Bhatt, Alannah Rivera-Cancel, Rishi Makkar, Philip A. Kragel, Thomas Rodriguez, John L. Graner, Anita Alaverdyan, Kareem Hamadani, Priten Vora, Bruce Naliboff, Jennifer S. Labus, Kevin S. LaBar, Emeran A. Mayer, Nancy Zucker

**Affiliations:** 1 G. Oppenheimer Center for Neurobiology of Stress and Resilience, UCLA, Los Angeles, CA 90095 USA; 2 David Geffen School of Medicine, UCLA, Los Angeles, CA 90095 USA; 3 Vatche and Tamar Manoukian Division of Digestive Diseases, UCLA, Los Angeles, CA 90095 USA; 4 Ahmanson-Lovelace Brain Mapping Center, UCLA, Los Angeles, CA 90095 USA; 5 Department of Psychiatry and Behavioral Sciences, Duke University, Durham, USA; 6 Department of Psychology and Neuroscience, Duke University, Durham, USA; 7 Imaging Genetics Center, Mark and Mary Stevens Institute for Neuroimaging and Informatics, Keck School of Medicine at USC, University of Southern California, Los Angeles, USA; 8 Department of Psychology, Emory University, Atlanta, USA

**Keywords:** Anorexia nervosa, Network metrics, Sensorimotor network, Basal ganglia, Functional connectivity

## Abstract

**Background:**

Anorexia nervosa (AN) is a disorder characterized by an incapacitating fear of weight gain and by a disturbance in the way the body is experienced, facets that motivate dangerous weight loss behaviors. Multimodal neuroimaging studies highlight atypical neural activity in brain networks involved in interoceptive awareness and reward processing.

**Methods:**

The current study used resting-state neuroimaging to model the architecture of large-scale functional brain networks and characterize network properties of individual brain regions to clinical measures. Resting-state neuroimaging was conducted in 62 adolescents, 22 (21 female) with a history of AN and 40 (39 female) healthy controls (HCs). Sensorimotor and basal ganglia regions, as part of a 165-region whole-brain network, were investigated. Subject-specific functional brain networks were computed to index centrality. A contrast analysis within the general linear model covarying for age was performed. Correlations between network properties and behavioral measures were conducted (significance q < .05).

**Results:**

Compared to HCs, AN had lower connectivity from sensorimotor regions, and greater connectivity from the left caudate nucleus to the right postcentral gyrus. AN demonstrated lower sensorimotor centrality, but higher basal ganglia centrality. Sensorimotor connectivity dyads and centrality exhibited negative correlations with body dissatisfaction and drive for thinness, two essential features of AN.

**Conclusions:**

These findings suggest that AN is associated with greater communication from the basal ganglia, and lower information propagation in sensorimotor cortices. This is consistent with the clinical presentation of AN, where individuals exhibit patterns of rigid habitual behavior that is not responsive to bodily needs, and seem “disconnected” from their bodies.

**Supplementary Information:**

The online version contains supplementary material available at 10.1186/s40337-022-00534-9.

## Plain English summary

Individuals with anorexia nervosa (AN) usually report a fear of gaining weight. They often develop a dislike and distrust of their bodies, feeling that their bodies had somehow let them down. These fears can in turn lead to dangerous weight loss behaviors. Magnetic resonance imaging of the brain is a tool that helps highlight the underlying biological processes associated with AN. In the current study we aim to investigate how the connections in key regions of the brain are related to clinical and behavioral factors associated with AN. We found regions of two main networks were associated with body dissatisfaction and drive for thinness, which are key features of AN. The brain regions involved help explain why patients with AN have characteristics of feeling disconnected from their bodies, having difficulty labeling and regulating emotions, responding to biological needs such as hunger and fatigue, and differentiating experiences that will be rewarding. These results can help guide interventions that will be directed towards helping individuals with AN to better sense, decipher, and act on the various signals being communicated by their body.

**Supplementary Information:**

The online version contains supplementary material available at 10.1186/s40337-022-00534-9.

## Introduction

Central to the disorder of anorexia nervosa (AN) is severe weight loss and persistent low-body weight that radically alters typical trajectories of adolescent social and emotional development [1]. Adolescent girls and young adult women are at the greatest risk for AN [2], with evidence indicating that the incidence of AN is emerging at younger ages than prior generations [3]. Fear of weight gain is the diagnostic feature intended to encapsulate the motivation for the intractable drive to lose weight in AN [4]. Compounding this fear is a diagnostic feature described as disturbance in the experience of the body, a feature proposed to be constituted of difficulties with the adaptive integration of interoceptive [5, 6] or proprioceptive [7] cues or with problems of multi-sensory integration more broadly [8]. Critically, body image disturbance in AN is a particularly intractable feature, one that have been found to persist following weight restoration. Advances in our understanding of neural circuit function, particularly in those circuits implicated in the experience of the body, such as the sensorimotor cortex and basal ganglia, may help us to understand the constituents of body image experience and the relentless drive for thinness in AN across the spectrum of weight loss and restoration.

Structural and functional alterations in the sensorimotor network have been consistently reported in patients with AN [9] and have been associated with visuomotor disturbances [10] and deficits in spatial organization [11]. Compared to healthy controls (HCs), individuals with AN have lower gray matter volume in the sensorimotor network [12]. Using a region-of-interest-based approach, it has been shown that individuals with AN have lower functional connectivity between the sensorimotor and visual networks, suggesting that altered visuospatial processing may be related to body image disturbances [10]. Using a network-based statistic approach, it has been shown that a sub-network of regions including the thalamus and posterior insula had lower connectivity compared to HCs, indicating that sensory information may be propagated unreliably or inefficiently [13]. Finally, a seed-based approach found abnormal thalamocortical connectivity that was associated with deficits in cognitive-control tasks in AN [14], revealing potential neurobiological mechanisms underlying cognitive function. When viewed together, such findings may help to explain the discrepancy between individuals’ actual and perceived body state.

Dysregulation in serotonin and dopamine circuits in the basal ganglia may play a role in the hyperactivity of motivational systems - which (may) contribute to the desire to suppress appetitive systems associated with symptoms of AN [15–19]. Increased resting state functional connectivity between the nucleus accumbens and orbital frontal gyrus, and increased structural connectivity, measured via diffusion tensor imaging, has been associated with elevated scores on a self-report measure of eating disorder symptoms in individuals with AN aged 16 to 25 years old [20]. Greater ventral striatal activity in individuals with AN has been observed in response to visual stimuli of under-weight vs normal weight individuals compared to HCs and vise-versa [21], supporting the rewarding value of visual stimuli related to starvation. Similar findings have also been reported in adolescents [22], a response hypothesized to reflect maladaptive conditioning: the reinforcing value of cues associated with food restriction.

Despite the growing understanding of the specific involvement of brain networks in AN, most studies have focused on individual brain regions, and a deeper understanding of the underlying properties and architecture of key brain networks is not fully understood. Graph theory has been used to characterize brain regions, their connections, and the integrity and information flow of brain networks. Measures of *centrality* are common measures of global connectivity that participate in integrative processing and associated behavioral responses [23–25]. Regions with high centrality are influential in communicating and facilitating flow of information with other regions in the brain network and have greater resilience to insult [23]. As centrality accounts for the relationship of the brain region’s properties with the entire functional connectome, brain regions with greater functional centrality indicate a greater amount of influence on the functional performance of the network [25]. Some recent work has been done investigating the structural [26] and functional [27–30] connectomes using graph theory approaches, and a recent review has shown nodal topological differences in brain regions including the insula, thalamus, basal ganglia, posterior occipital cortex, prefrontal cortex, inferior frontal gyrus, and precentral gyrus have been altered [31].

The aim of the current study was to quantify differences in resting-state functional connectivity and centrality in the sensorimotor and basal ganglia networks between individuals with anorexia nervosa (AN) and HCs. We aimed to test the hypothesis that individuals with AN demonstrate lower connectivity and centrality in core regions of sensorimotor networks, and greater connectivity in the basal ganglia networks compared to HCs, and that these differences would be associated with differences in eating disorder symptoms. Specifically, we hypothesized that lower connectivity and centrality in sensorimotor networks would be associated with increased body dissatisfaction, an evaluative measure of body image disturbance. We further hypothesized that greater connectivity and centrality in the basal ganglia networks would be associated with greater drive for thinness, an index of the valence and intensity of threats to weight loss.

## Methods and materials

### Overview

Adolescents between the ages of 10–20 years old were recruited for a study of “gut feelings”. Recruited individuals participated in a laboratory session and a resting-state fMRI scan as a part of a larger study (reported elsewhere, see [32] and for details about these MRI tasks) [33]. Here we focus on the functional resting-state connectivity and functional network architecture from these scans.

### Recruitment

We aimed to recruit all individuals with AN who presented to a specialized outpatient clinic for the treatment of eating disorders at a Southeastern academic medical center. The control sample was recruited from this same medical clinic. Additional recruitment of both the clinical and HC samples was conducted throughout the university associated with this medical center. All participants under the age of 18 had informed consent from a parent and/or legal guardian.

#### Healthy control recruitment

For our control sample, we recruited from a pediatric primary care practice that was part of a Southeastern academic medical center (see Franz et al. [34] for general screening strategy adapted for this study). The demographic composition of this practice paralleled that of the surrounding county, and thus helped to facilitate the recruitment of a representative control group. Screening occurred on random weekdays.

#### Clinical recruitment

Clinical participants were also recruited within that medical practice. Recruiters attended every clinic session of pediatricians who were part of a specialized outpatient eating disorder program from the period of 9/1/2009–8/31/2011 to identify and screen all eligible AN participants, whether they currently met criteria for AN or had a history of AN and were attending a medical follow-up appointment.

#### Inclusion criteria

Study participants were required to have a current or prior diagnosis of AN consistent with symptoms delineated in the Diagnostic and Statistical Manual of Mental Disorders, 5th edition (13). Medications were permitted provided the individual was on a stable dose for a period $\ge$ 3 months. See Additional file 1: Table S1 for medication list.

**Table 1:** Study demographics and clinical behavioral measures

	Anorexia nervosa (n = 22)	Healthy controls (n = 40)	t-value	*p*-value	Cohen’s D
**Sample descriptive: mean (Std); range**					
Age in Years	17.4 (2.3); 12.3–19.9	15.1 (3.1); 10.8–20.0	3.05	0.004	0.84
Body Mass Index (BMI, kg/m^2^)	20.2 (2.5); 15.1–26.6^a^	23.2 (5.2); 15.8–39.3^b^	− 2.54	0.04	− 0.74
z-BMI	− .43 (.87); − 2.19–0.75	.60 (1.03); − 1.49 − 2.58^b^			
Age-adjusted Weight Percentile	38.9 (24.9); 1–77	66.0 (28.4); 7–99^b^			
Sex					
Female	21 (95.5%)	39 (97.5%)			
Male	1 (0.5%)	1 (2.5%)			
Race: Count (Percentage)					
White	18 (81.8%)	19 (47.5%)			
Black	1 (4.5%)	16 (40.0%)			
Asian	2 (9.1%)	2 (5%)			
Other	1 (4.5%)	3 (7.5%)			
**Disorder parameters: mean (Std); range**					
Age of Onset in Years	13.4 (1.8); 10–17	n/a			
Time to Treatment in Months	12.6 (10.9); 0–36	n/a			
Length of Illness in Months	36.3 (23.6); 3–96	n/a			
Time at Unhealthy Low Weight in Months	12.3 (11.2); 3–40	n/a			
Current Disorder Status: Count (Percentage)^c^					
Acute AN Partially-Weight Restored AN Fully Weight-Restored AN	2 (9.1%) 3 (13.6%) 17 (77.3%)				
**Self-Report Symptom Measures**^**d**^**: Mean (Std), Range**					
Drive for Thinness	15.05(7.9); 0–24	4.18 (5.3); 0–20	6.47	< 0.0001	1.62
Body Dissatisfaction	15.21 (7.5); 0–28	4.53 (5.0); 0–17	6.71	< 0.0001	1.68
Bulimia	3.79 (5.5); 0–19	1.41 (1.7); 0–5	2.54	0.01	0.58
Perfectionism	12.47 (6.5); 3–24	13.65 (5.8); 5–24	− 0.73	0.47	0.19
Total Score	46.53 (19.24); 4–79	23.75 (12.61); 5–53	4.34	1.41e − ^4^	1.41

Questionnaires: Body Mass Index (BMI), Age Onset, Length Illness, Time TX, Age Illness End, EDI Bulimia, EDI Body Dissatisfaction, EDI Drive for Thinness, EDI Perfectionism

*N*: subject number, *SD* standard deviation, *AN* Anorexia Nervosa, *HC* Healthy Controls, *EDI* Eating Disorder Inventory, *TX* Treatment

^a^The sample reflects individuals with a history of anorexia nervosa or atypical anorexia nervosa (in this case, a lose of 25% of body weight including crossing two weight percentiles, but being above a designated underweight BMI at baseline) at various stages of weight restoration

^b^Two individuals in the typical control group had very low weight. However, a review of their medical chart, maternal report, and self-report measures revealed no signs of an eating disorder and there was no evidence of physical symptoms that may be present if an individual was underweight (e.g., bradycardia)

^c^ENIGMA definitions for weight status in combination with medical chart evidence, maternal report, and self-report measures of eating disorder symptoms

^d^Subscales from the Eating Disorder Inventory, 3rd edition

#### Exclusion criteria

Adolescents were excluded if either they or their mother did not have fluency in English, had an IQ < 70, failed to meet MRI safety requirements [35–37], were suicidal, exhibited symptoms of psychosis, or actively abused substances. In addition, healthy control participants could not have a history of an eating disorder or currently meet criteria for a psychiatric diagnosis as determined by screening for current symptoms (see below) and parent and participant report.

### Procedures

#### Overview

Adolescents and their parents attended an initial laboratory session during which diagnostic information was obtained. The adolescent participated in a mock scanning session to familiarize themselves with the scanning environment and to obtain training in procedures that would maximize the amount of usable scanning data (e.g., teaching to minimize movement). Height and weight were obtained at the time of scanning. Individuals on medications with short half-lives (e.g., stimulant medication) were asked to refrain from taking medication on the day of scanning.

#### Consent

Written informed consent was obtained from parents and participants above the age of 18, assent was obtained from participants from age 10 up to 18 years. The study was approved by the Institutional Review Board at Duke University Medical Center, and all methods were carried out in accordance with relevant guidelines and regulations from the Declaration of Helsinki.

### Assessments

#### Screening

HC participants were screened for the absence of mental health symptoms using questions used to predict diagnostic status from a prior population cohort study of child and adolescent’s psychopathology [34]. Children who scored above the screen cut-off were excluded from further participation but were given a small prize.

#### Determination of diagnosis and diagnostic history

We attempted to recruit all individuals with a history of AN who presented at an outpatient medical clinic. These individuals were at various stages of the disorder in terms of degree of weight severity or restoration, and thus the sample had significant heterogeneity. While this had the disadvantage of preventing comparisons between categorical stages of the disorder, it had the advantage of high external validity in that this group reflected individuals presenting for care. We describe individuals dimensionally and categorically. For both, diagnosis and parameters of illness history were determined by systematically combining several sources of data: 1) maternal report of her child’s illness history; 2) adolescent completion of self-report measures of current symptoms; 3) adolescent report of illness history; and 4) medical chart abstraction. This included both BMI and zBMI (i.e., age-adjusted BMI, which accounts for height, weight, and age) and age-adjusted weight percentile. To be classified as a HC, 1) parent report indicated no history of an eating disorder; 2) adolescent self-report of Drive for Thinness values were within 1 standard deviation of normative values; and 3) the medical chart contained no reference to an eating disorder diagnosis.

For the clinical group, we employed the ENIGMA Eating Disorders consortium definitions of weight status to define individuals with AN that were currently ill or partially weight-restored. These definitions were complemented with parent report of disorder history and self-reports of Drive for Thinness as described below. The ENIGMA consortium (http://enigma.ini.usc.edu/about-2/) is an international effort combining data across research sites to accelerate the study of health and disease across development. ENIGMA definitions of weight status are employed in this manuscript for ease of comparison across studies. To these definitions of weight status, we added benchmarks for scores on eating disorder measures as defined below. Acute AN (AN) is a BMI of ≤ 17.5 kg/m^2^, < 10th for weight according to age-adjusted weight-percentile, and not in a period of rapid weight gain (< 2 kg. over the past 4 weeks). This weight definition was complemented with the following definitions for AN in this study: 1) parent records indicated the child had AN within past 3–6 months; 2) medical chart had a diagnosis of AN; and 3) the adolescent had a Drive for Thinness score > 1 std above normative values. For partially weight-restored AN (ANp), according to ENIGMA: participant does not meet criteria for acute AN and either: A) BMI is < 18.5 kg/m^2^ or < 10th adjusted percentile; or B) BMI is > 18.5 kg/m^2^ but < 19.5 kg/m^2^, age-adjusted percentile is > 10th but < 25th, participant must not have regular menses, and still show significant eating disorder symptoms as defined in this study as > 1 standard deviation of Drive for Thinness Normative Values. In this study, weight-restored AN (ANwr), was defined as: 1) BMI ≥ 18.5 or the parent report indicated that the child was without an eating disorder for 3–6 months; 2) the medical chart review did not contain a current diagnosis of AN and 3) there was no evidence of a medical sign that weight was low (e.g., bradycardia, orthostatic hypotension). To determine length of illness, mothers were asked the age at which their child first developed an eating disorder, the type of eating disorder, and whether this diagnosis was verified by a health care professional. This information was compared and combined with the medical record and referenced against the child’s weight history, current weight, and current symptom endorsement. In only one case was there a discrepancy. In this case, the parent indicated that the child no longer had an eating disorder and had been at a healthy weight for 3–6 months. However, the child’s weight and endorsement of clinical symptoms were both above clinical cut-offs. Of interest, this child had a long duration of illness (> 7 years) and a lowest BMI of 11. Her current BMI of 18, may have seemed to present as significant progress (as it was), yet an anchor of normality had been lost.

A similar strategy was employed to determine months of weight restoration. Parents were asked the length of time the child had been at a healthy weight and this was verified relative to the child’s weight history and medical record. Again, there was one discrepancy, noted below.

#### Self-report measures

The Eating Disorder Inventory (3rd Edition) is one of the most widely used measures of eating disorder symptomatology and associated features [38]. This measure was used to characterize the sample relative to other studies and provide a continuous index of current symptoms. Three subscales that measure the core pathology of eating disorders were administered in the current sample: Drive for Thinness, Bulimia, and Body Dissatisfaction. All scales have extensive validity and reliability information as well as normative data from clinical and non-clinical samples. The Drive for Thinness subscale is a 7-item scale that assesses “an extreme desire to be thinner, preoccupation with weight, and an intense fear of weight gain”. Extensive reliability, construct, and predictive validity have been established [39–41]. The internal consistency of this scale was measured via Cronbach’s alpha, which is a measure of internal consistency (between 0 and 1), or how closely related a set of items are as a group. High values indicate high reliability [42]. Cronbach’s alpha for the Drive for Thinness subscale in our sample was α = 0.95. The Bulimia subscale is an 8-item scale used to index the tendency to think about or engage in uncontrollable overeating or eating in response to emotions. The internal consistency in our sample was α = 0.89. The Body Dissatisfaction subscale is a 7-item scale that assesses discontentment with the size and shape of various body parts that are of particular concern to those with eating disorders (e.g., stomach). The internal consistency in our sample was α = 0.94. We also looked at the Perfectionism subscale of the EDI, a 6-item scale that evaluates the personal value that individuals place on personal achievement and meeting their own high standards. The internal consistency in our sample was α = 0.91.

### MRI acquisition and quality control

Whole brain structural and functional (resting state) data was acquired using MRI scans conducted on a 3 Tesla General Electric MR 750 system with 50-mT/m gradients and an 8-channel head coil for parallel imaging (General Electric, Waukesha,WI, USA). Noise reducing headphones were used. To control for the state of acute nourishment on brain activity parameters [43], individuals were asked to fast for 2 h prior to the scan and then were asked to consume a small, standardized snack just prior to the scan [43]. Twelve of the participants were on medication the day of scanning.

#### Structural gray-matter

For registration purposes, a high-resolution structural image was obtained from each subject using a magnetization-prepared rapid acquisition gradient-echo sequence in the axial plane (Ax FSPGR BRAVO, repetition time = 7.58 ms, structural acquisition time = 3 min 22 s, echo time = 2.936 ms, inversion time = 450 ms, flip angle = 12°, slice thickness = 1 mm, 256 slices, 256 × 256 voxel matrix, 1 mm voxel size).

#### Resting state functional connectivity

Resting state fMRI data was acquired using the following parameters: (34-slice, 150 whole brain volumes, interleaved slices, slice thickness = 3.8 mm, TE: 30 ms, TR 2000 ms, resting-duration (TA) = flip angle = 70°, acquisition matrix = 64 × 128, field of view = 243 mm × 243 mm). Subjects rested with eyes open and instructed to fixate on a cross while functional blood oxygen-level dependent images were acquired.

### MRI processing

#### Resting state functional connectivity pre-processing

Resting state preprocessing was conducted using SPM12 software (Welcome Department of Cognitive Neurology, London, UK). The first two volumes were discarded to allow for stabilization of the magnetic field. Slice timing correction was performed first, followed by rigid six-degree motion-correction realignment. The motion correction parameters in each degree were examined for excessive motion. If any volume-to-volume motion correction parameter was above 2 mm translation or 2° rotation, it was excluded from the dataset. To robustly take account of the effects of motion, root mean squared (RMS) realignment estimates were calculated as robust measures of motion using publicly available MATLAB code from GitHub [44]. Any subjects with a RMS value greater than 0.25 were not included in the analysis [44]. No participants had a RMS value greater than 0.25. The resting state images were then co-registered to their respective anatomical T1 images. Each T1 image was then segmented and normalized to a smoothed template brain in 2 mm Montreal Neurological Institute (MNI) template space. Each subject's T1 normalization parameters were then applied to that subject's resting state image, resulting in an MNI space normalized resting state image. The resulting images were smoothed with a 5 × 5 × 5 mm^3^ FWHM Gaussian kernel. For each subject, a sample of the volumes was inspected for any artifacts and anomalies. Levels of signal dropout were also visually inspected for excessive dropout in a priori regions of interest.

##### Functional network connectivity construction

Preprocessed and normalized functional images were entered into the CONN-fMRI functional connectivity toolbox version 17 [45]. Regions from the Destrieux [46] and Harvard–Oxford Subcortical Atlases were entered as ROIs. These atlases were used to accurately capture the ROIs mentioned in the previous research. CompCor, a component-based noise correction method, was applied to remove physiological noise without regressing out the global signal [47]. White-matter, cerebrospinal fluid, six realignment parameters, and first-order temporal derivatives of motion, and RMS were removed using regression. This ensures only signal from gray matter voxels are being considered. Band pass filtering between 0.01 and 0.08 Hz was applied to the residualized time series to reduce the low- and high-frequency noises after regression. Linear measures of ROI-to-ROI functional connectivity were computed using Fisher transformed correlations representing the association between average temporal BOLD time series signals across all voxels in a brain region. The final outputs for each subject consisted of a 165 × 165 matrix consisting of Fisher transformed Z correlation values between each ROI. Overall functional connectivity was computed by taking the mean of all positive values in each individual’s matrix. This was done to determine if proportional thresholding should be used, as because a minimal difference in overall FC can cause a difference in network metrics, which may be due to inherent disease differences [48]. An independent sample t-test was done to determine if there was a significant difference in overall FC between groups.

##### Computing network metrics

The Graph Theory GLM toolbox (GTG) (http://www.nitrc.org/projects/metalab_gtg) and in-house MATLAB scripts were applied to the subject-specific functional brain networks to compute two local weighted network metrics indexing centrality. We decided to focus on centrality at the microscale level as this allows one to determine characteristic hub roles for specific nodes, or brain regions, which can be easily interpretable by scientists and clinicians alike [49]. Additionally measures of centrality can capture a node’s role in network organization beyond local connections [49]. Measures of centrality quantify the importance of a region’s influence on communication and information flow in large-scale brain networks [25]. These measures include strength and betweenness centrality [23]. Strength represents the weighted sum of the number of connections a given brain region has and reflects a brain region’s total impact in the network [23]. Betweenness centrality describes the degree to which a brain region lies on the shortest path between two other regions [23]. Acting as way stations, regions with high betweenness centrality are topologically primed to control communication between other regions. The magnitude of the Z values represents the weights in the functional network. The Z values in each individual connectivity matrix was thresholded at Z > 0.3, and all other values were set to zero. A threshold of 0.3 was chosen since a correlation of 0.3 represents a medium effect size, and the inclusion of lower correlations could result in the inclusion of less accurate estimates [50]. We did not use a proportional-based thresholding approach because minimal differences in overall functional connectivity may introduce group differences in network metrics in patient vs. control studies [48]. All visualizations were created using in-house visualization schematics along with the BrainNet Viewer [51].

### MRI data analysis

#### Regions of interest

Many of our analyses were based on regions of interest (ROI). For comparison between the combined AN group, consisting of both ANC and ANR individuals, and the HC group, core ROIs of the sensorimotor and basal ganglia networks were examined in relationship to the entire brain parcellated by the Destreiux [46] cortical and Harvard–Oxford subcortical [52–55] atlases, as well as seed-to-voxel whole brain analyses (Additional file 1: Table S2, Fig. S1). Core regions of the *sensorimotor network* included the thalamus [Tha], brain stem [Bstem], hippocampus [Hip], paracentral lobule and sulcus [PaCL/S], primary somatosensory cortex [S1], central sulcus [CS], primary motor cortex [M1], precuneus [PrCun], secondary somatosensory cortex [S2], supplementary motor area [M2], middle insula [part of aINS], and posterior insula [pINS] [10, 11]. Core regions of the *basal ganglia network* included the basal ganglia [BG] and globus pallidus [Pal] [56]. These core seed ROIs were selected from past research in AN and used to look at differences throughout ROIs across the entire brain parcellated by the Destreiux [46] cortical and Harvard–Oxford subcortical [52–55] atlases, as well as seed-to-voxel whole brain analyses.

**Table 2:** Seed-to-voxel results from the sensorimotor and basal-Ganglia networks

Seed	Region	X	Y	Z	k (cluster size)	t-value	*p*-value	q -value	β value	Interpretation
**Sensorimotor network (AN vs. HC)**										
**to sensorimotor network**										
Right superior frontal gyrus	Right precentral gyrus	40	− 26	60	306	− 5.92	0.000008	< 0.000001	− 0.21	AN < HC
Right paracentral lobule and sulcus	Right postcentral gyrus & right precentral gyrus	40	− 20	52	281	− 5.53	< 0.000001	< 0.000001	− 0.25	AN < HC
Left central sulcus	Right supramarginal gyrus, posterior division	60	− 38	20	128	− 5.43	0.000004	0.0001	− 0.24	AN < HC
Right central sulcus	Right supramarginal gyrus, posterior division	2	− 14	66	96	− 5.4	0.000015	0.001	− 0.24	AN < HC
Left postcentral gyrus	Right supramarginal gyrus, superior division	66	− 46	26	133	− 5.34	0.000009	0.00007	− 0.21	AN < HC
Left precentral gyrus	Right supramarginal gyrus, posterior division	64	− 48	28	88	− 5.32	0.000004	0.002	− 0.22	AN < HC
Right postcentral gyrus	Right supramarginal gyrus, superior division	58	− 42	32	227	− 5.27	0.000002	< 0.000001	− 0.23	AN < HC
Left paracentral lobule and sulcus	Right precentral gyrus	40	− 20	52	139	− 5.09	0.000001	0.00005	− 0.25	AN < HC
Left central sulcus	Right precentral gyrus	10	− 18	68	69	− 5.02	0.000025	0.006	− 0.23	AN < HC
Left central sulcus	Left superior frontal gyrus	0	42	38	53	− 4.99	0.000036	0.017	− 0.25	AN < HC
Left superior part of the precentral sulcus	Left precentral gyrus	28	− 24	58	152	− 4.94	0.000035	0.00002	− 0.23	AN < HC
Left long insular gyrus and central sulcus of the insula	Left postcentral gyrus	− 42	− 30	46	37	− 4.9	0.00001	0.019	− 0.2	AN > HC
Left precentral gyrus	Left precentral gyrus	4	− 38	56	68	− 4.82	0.000016	0.005	− 0.24	AN < HC
Left superior frontal gyrus	Left precentral gyrus	− 36	− 20	46	58	− 4.8	0.000039	0.012	− 0.2	AN < HC
Left central sulcus	Left superior frontal gyrus	0	36	52	51	− 4.75	0.000016	0.017	− 0.2	AN < HC
Left hippocampus	Right supramarginal gyrus, posterior division	60	− 38	22	95	− 4.72	0.000012	0.0007	− 0.21	AN < HC
Right hippocampus	Left supramarginal gyrus, posterior division	− 60	− 40	44	48	− 4.72	0.000016	0.036	− 0.2	AN < HC
Left superior part of the precentral sulcus	Left postcentral gyrus	12	− 34	68	125	− 4.67	0.000013	0.00006	− 0.22	AN < HC
Right superior frontal gyrus	Left postcentral gyrus	− 38	− 34	46	48	− 4.67	0.000029	0.0006	− 0.21	AN < HC
Left central sulcus	Left precentral gyrus	− 6	− 24	56	40	− 4.65	0.000087	0.034	− 0.21	AN < HC
Right central sulcus	Right supramarginal gyrus, posterior division	58	− 36	22	84	− 4.65	0.000028	0.002	− 0.23	AN < HC
Left posterior ramus (or segment) of the lateral sulcus (or fissure)	Right precentral gyrus	22	− 22	60	43	− 4.48	< 0.000001	0.001	− 0.21	AN < HC
Left superior part of the precentral sulcus	Left precentral gyrus	− 4	− 20	70	59	− 4.45	0.000008	0.009	− 0.22	AN < HC
Left superior part of the precentral sulcus	Left precentral gyrus	− 50	− 14	48	51	− 4.41	0.000117	0.014	− 0.23	AN < HC
**to default mode network**										
Left posterior ramus (or segment) of the lateral sulcus (or fissure)	Right angular gyrus	56	− 46	28	211	− 5.97	0.000004	< 0.000001	− 0.22	AN < HC
Left postcentral sulcus	Right temporal pole	46	14	− 34	51	− 5.29	0.000001	0.032	− 0.21	AN < HC
Right subcentral gyrus (central operculum) and sulci	Left cingulate gyrus, posterior division	− 62	− 58	30	54	− 5.1	0.000011	0.024	− 0.23	AN < HC
Left superior frontal gyrus	Right angular gyrus	58	− 56	36	105	− 4.92	0.000003	0.0004	− 0.24	AN < HC
Left posterior ramus (or segment) of the lateral sulcus (or fissure)	Right middle temporal gyrus, posterior division	54	− 30	− 8	50	− 4.89	0.000029	0.0006	− 0.23	AN < HC
Right superior segment of the circular sulcus of the insula	Cingulate gyrus, posterior division	0	− 28	34	104	− 4.89	0.000074	0.0006	− 0.21	AN < HC
Left thalamus	Right angular gyrus	58	− 50	28	93	− 4.88	0.000347	0.017	− 0.21	AN < HC
Right thalamus	Right angular gyrus	58	− 50	30	65	− 4.88	0.000123	0.004	− 0.19	AN < HC
Right inferior part of the precentral sulcus	Right superior temporal gyrus, posterior division	62	− 18	0	41	− 4.85	0.000012	0.048	− 0.23	AN < HC
Right superior part of the precentral sulcus	Left precuneus cortex	− 12	− 66	20	118	− 4.59	0.000016	0.0003	− 0.24	AN < HC
Right subcentral gyrus (central operculum) and sulci	Right cingulate gyrus, posterior division	4	− 28	42	50	− 4.58	0.000099	0.024	− 0.19	AN < HC
Right superior part of the precentral sulcus	Right precuneus cortex	8	− 60	16	70	− 4.52	0.0000093	0.006	− 0.22	AN < HC
Right subcentral gyrus (central operculum) and sulci	Right angular gyrus	64	− 50	28	48	− 4.45	0.000004	0.024	− 0.24	AN < HC
Right long insular gyrus and central sulcus of the insula	Left angular gyrus	− 62	− 54	32	51	− 4.3	0.000033	0.020	− 0.25	AN < HC
Left inferior segment of the circular sulcus of the insula	Cingulate gyrus, posterior division	0	− 26	36	67	− 4.19	0.000103	0.011	− 0.21	AN < HC
**to emotion regulation network**										
Right hippocampus	Right inferior frontal gyrus, pars opercularis	48	14	18	129	− 5.92	0.000005	0.00007	− 0.19	AN < HC
Right postcentral gyrus	Left inferior frontal gyrus, pars triangularis	− 48	30	− 4	59	− 5.07	0.000024	0.013	− 0.2	AN < HC
Left postcentral sulcus	Left inferior frontal gyrus, pars triangularis	− 54	20	20	44	− 4.86	0.000005	0.043	− 0.21	AN < HC
Left central sulcus	Left paracingulate gyrus	− 6	46	12	38	− 4.65	0.000005	0.036	− 0.2	AN < HC
**to central executive network**										
Left postcentral sulcus	Left middle frontal gyrus	− 42	6	48	71	− 6.19	0.000012	0.010	− 0.2	AN < HC
**to occipital network**										
Left central sulcus	Left lateral occipital cortex, superior division	− 18	− 76	32	43	− 4.72	0.000022	0.030	− 0.19	AN < HC

Seed	Region	X	Y	Z	k	t	p-value	q -value	β value	Interpretation
**basal ganglia network (AN vs. HC)**										
**to sensorimotor network**										
Left caudate nucleus	Right postcentral gyrus	68	− 12	28	46	5.07	0.000017	0.039	0.16	AN > HC
**to default mode network**										
Brainstem	Right angular gyrus	60	− 50	28	85	− 5.68	0.000008	0.002	− 0.19	AN < HC

Groups: Anorexia Nervosa (AN), Healthy Controls (HC)

*AN* Anorexia Nervosa, *HC* Healthy Controls

X Y Z coordinates are located in MNI Space. k: cluster size, q-value: FDR corrected *p*-value, β: beta

**Fig. 1:**
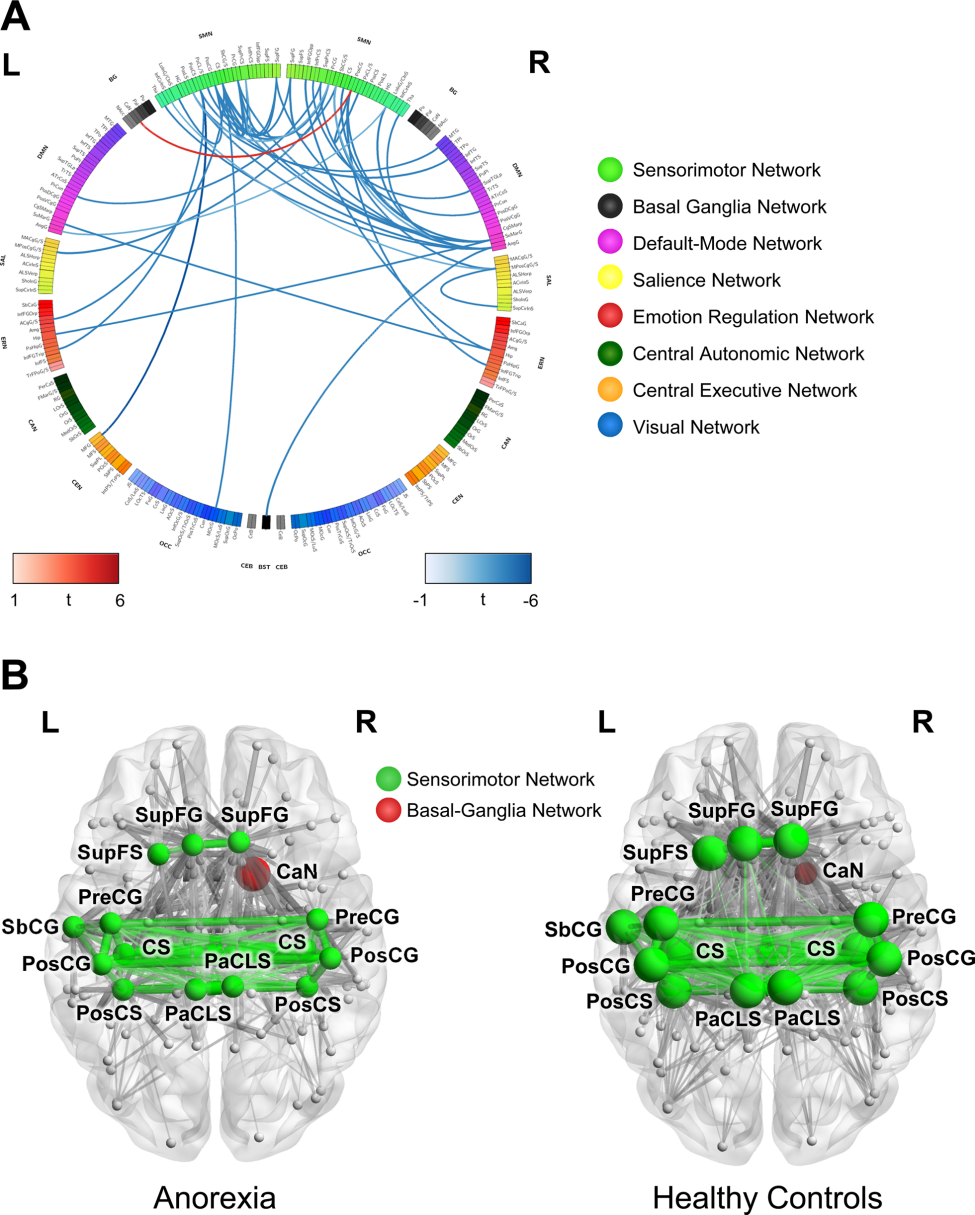
**A** Differences in functional connectivity between anorexia and healthy controls using whole-brain seed-to-voxel analysis: *SMN* sensorimotor network, *BG* Basal Ganglia, *DMN* default mode network, *SAL* salience network, *ERN* emotion regulation network, *CAN* central autonomic network, *CEN* central executive network, *Occ* occipital network; *BST* Brainstem Blue: Anorexia < Healthy Controls, Red: Anorexia > Healthy Controls t: t-score. **B** Differences in functional centrality between anorexia and healthy controls in the sensorimotor and basal ganglia networks: *CaN* caudate nucleus, *SupFG* superior frontal gyrus, *SupFS* superior frontal sulcus, *SubCG* subcentral gyrus and sulcus, *PreCG* precentral gyrus, *PosCG* postcentral gyrus, *PosCS* postcentral sulcus, *CS* central sulcus, *PaCLS* paracentral lobule and sulcus. Node size represents the degree of centrality of that node based on resting-state functional connectivity. Edge thickness represents the correlation of connectivity between two nodes. Edges between two brain regions in the sensorimotor network are colored in green.

#### Functional seed-to-voxel whole-brain analysis

In order to determine differences in whole brain connectivity from selected ROIs, we performed a whole brain, seed-to-voxel analysis in CONN utilizing the GLM and controlling for age. This represents the level of functional connectivity between each ROI and every voxel in the brain. The parametric map of t-values were thresholded using an initial height threshold (voxel-level) of *p* < 0.001 and corrected (using the false discovery rate method); cluster thresholds were set at *p*_*(FDR)*_ < 0.05 [57]. In order to perform partial correlations controlling for age with behavioral variables, eigenvalues for each connectivity unit (i.e., the degree of connectivity between the seed and significant cluster of voxels) were extracted from within the CONN toolbox. Significance was set at *p* < 0.05. Visualizations were done using circus [58] in Linux.

#### Computing group differences in network metrics

In order to test for disease-related differences, a GLM was applied and the effect of age was included as a covariate in the model. Significance was determined via Freedman & Lane’s non-parametric permutation testing strategy and specifying 10,000 permutations [59]. This method provides good control over type I error rates and is robust to the presence of outliers [60]. Probability values from the permutation testing strategy were corrected using a false discovery rate (FDR) adjusted *p*-value, where q < 0.05 was considered significant [61, 62]. FDR correction was applied at the whole-brain level. Partial correlations controlling for age were then computed to determine the association between significant network metrics and behavioral measures. Significance was set at *p* < 0.05.

### Behavioral/clinical data

Group differences in clinical and behavioral measures were evaluated by applying linear contrast analyses in a GLM model using Statistical Package for the Social Sciences (SPSS) software (version 22). To quantify the differences between the various contrasts, we calculated Cohen’s effect size *d*, reflecting differences on the scale of standard deviation units, where values are interpreted as low (*d* = 0.20), moderate (*d* = 0.50), and high (*d* = 0.80) [63]. Correlations between significant findings for group differences in connectivity and measures of centrality were conducted with behavioral variables, while controlling for age.

## Results

### Demographics and clinical variables

AN participants had lower BMI compared to HCs (*t*_*(60)*_ =  − 2.54, *p* = 0.01, *d* =  − 0.74), and were older (*t*_*(60)*_ = 3.05, *p* = 0.004, *d* = 0.84). AN participants had greater scores on the EDI Body Dissatisfaction subscale (*t*_*(60)*_ = 6.71, *p* < 0.0001, *d* = 1.68), EDI Drive for Thinness subscale (*t*_*(60)*_ = 6.47, *p* < 0.0001, *d* = 1.62), EDI Bulimia subscale (*t*_*(60)*_ = 2.54, *p* = 0.01, *d* = 0.58), and EDI Total scores (*t*_*(30.74)*_ = 4.34, *p* = 1.41 × 10^−4^, *d* = 1.41), but not the EDI Perfectionism subscale (*t*_*(60)*_ =  − 0.73, *p* = 0.47, *d* = 0.19). See Table 1 for demographic details.

### Overall functional connectivity

Overall functional connectivity comparing anorexia (mean = 0.16, SD = 0.040) and healthy controls (mean = 0.23, SD = 0.095) was significantly different (*t*_*(57.06)*_ =  − 3.92, *p* = 0.0002, *d* =  − 0.84).

### Disease related differences in seed-to-voxel whole brain resting-state functional connectivity

#### Sensorimotor network

Significant differences were observed looking at whole brain resting-state functional connectivity differences from seeds within the sensorimotor network (46 connectivity dyads, *β* ranging from − 0.19 to − 0.25, *q*-values ranging from 0.04 to 0.000007). All connectivity results resulted in AN participants having lower resting-state functional connectivity from sensorimotor regions to other brain areas compared to HCs. See Table 2 and Fig. 1A for details.

#### Basal ganglia network

AN participants had greater resting-state functional connectivity from the left caudate nucleus to the right postcentral gyrus (k (cluster size) = 46, *β* = 0.16, *t* = 5.07, *p*_*(FDR)*_ = 0.039), and lower resting-state functional connectivity from the brain stem to the right angular gyrus (k (cluster size) = 85, *β* =  − 0.19, *t* =  − 5.68, *p*_*(FDR)*_ = 0.002). See Table 2 and Fig. 1A for details.

### Disease related differences in measures of centrality

Significant disease-related differences in measures of centrality between AN and HC participants can be visualized in Table 3 and Fig. 1B. Subsequent analyses were restricted to regions significantly different between the AN and HC groups.

**Table 3:** Differences of functional network centrality in anorexia nervosa vs healthy controls

Region	*t*	*p*	*q*	*d*	Interpretation
**Basal ganglia network**					
**Betweenness centrality**					
Right caudate nucleus	3.24	0.006	0.02	0.63	ANC ↑ HC ↓
**Sensorimotor network**					
**Strength**					
Left paracentral lobule and sulcus	− 2.83	0.02	0.17	0.92	ANC ↓ HC ↑
Left subcentral gyrus and sulcus	− 2.9	0.02	0.14	0.68	ANC ↓ HC ↑
Left superior frontal gyrus	− 3.65	0.002	0.02	1.06	ANC ↓ HC ↑
Left postcentral gyrus	− 2.73	0.02	0.22	0.63	ANC ↓ HC ↑
Left precentral gyrus	− 3.07	0.01	0.09	0.75	ANC ↓ HC ↑
Left central sulcus	− 2.63	0.03	0.28	0.74	ANC ↓ HC ↑
Left superior frontal sulcus	− 3.24	0.006	0.06	0.99	ANC ↓ HC ↑
Left postcentral sulcus	− 2.67	0.03	0.25	0.7	ANC ↓ HC ↑
Right paracentral lobule and sulcus	− 2.97	0.01	0.12	0.83	ANC ↓ HC ↑
Right superior frontal gyrus	− 3.59	0.002	0.02	1.12	ANC ↓ HC ↑
Right postcentral gyrus	− 2.75	0.02	0.22	0.69	ANC ↓ HC ↑
Right precentral gyrus	− 3.02	0.01	0.1	0.73	ANC ↓ HC ↑
Right central sulcus	− 2.67	0.03	0.25	0.73	ANC ↓ HC ↑
Right postcentral sulcus	− 2.45	0.05	0.43	0.59	ANC ↓ HC ↑

*AN* Anorexia Nervosa, *HC* Healthy Controls; *t* t-value, *p* *p*-value, *q* FDR corrected *p*-value, *d* Cohen’s D, *PaCL*/*S* Paracentral lobule and sulcus, *SbCG*/*S* Subcentral gyrus and sulcus, *SupFG* Superior frontal gyrus, *PosCG* Postcentral gyrus, *PRCG* Precentral gyrus, *CS* Central sulcus, *SupFS* Superior frontal sulcus, *PosCS* Postcentral sulcus

#### Sensorimotor network

Subjects with AN had lower resting-state functional *strength* in the left (*t*_*(58)*_ =  − 3.65, *q* = 0.02, *d* = 1.06) and right (*t*_*(58)*_ =  − 3.59, *q* = 0.02, *d* = 1.12) superior frontal gyrus (SMA).

#### Basal ganglia network

Subjects with AN had greater resting-state functional *betweenness centrality* in the right caudate nucleus (*t*_*(58)*_ = 3.24, *q* = 0.02, *d* = 0.63).

### Correlations between brain analyses and behavioral variables

#### Whole brain seed-to-voxel analyses

Lower connectivity between the right postcentral gyrus and right supramarginal gyrus was associated with greater EDI Subscale Total scores (*r*_*(16)*_ =  − 0.759, *p* = 0.001, *q* = 0.03). Connectivity between the hippocampus and supramarginal gyrus was positively associated with time to treatment, and connectivity within the sensorimotor network was associated negatively with age of onset, however these correlations did not survive correction for multiple comparisons. See Table 4.

**Table 4:** Correlations between seed-to-voxel results, network metric results, and behavioral variables

Region 1	Region 2	Clinical variable	*r*	*df*	*p*-value	*q*-value
**Connectivity Dyad**						
**Sensorimotor network**						
Left postcentral gyrus	Right supramarginal gyrus—superior division	EDI Body Dissatisfaction	− 0.597	16	0.015	0.233
Left central sulcus	Right supramarginal gyrus—posterior division	EDI Body Dissatisfaction	− 0.562	16	0.023	0.280
Left superior part of the precentral sulcus	Left precentral gyrus	EDI Body Dissatisfaction	− 0.662	16	0.005	0.233
Right superior frontal gyrus	Right precentral gyrus	EDI Body Dissatisfaction	− 0.619	16	0.011	0.233
Left postcentral gyrus	Right supramarginal gyrus—superior division	EDI Drive for Thinness	− 0.564	16	0.023	0.273
Right postcentral gyrus	Right supramarginal gyrus—superior division	EDI Drive for Thinness	− 0.735	16	0.001	0.057
Left central sulcus	Right posterior supramarginal gyrus	EDI Drive for Thinness	− 0.587	16	0.017	0.270
Right superior part of the precentral sulcus	Right precuneus cortex	EDI Drive for Thinness	− 0.623	16	0.010	0.240
Left postcentral gyrus	Right supramarginal gyrus—superior division	EDI Subscale Total	− 0.533	16	0.034	0.201
**Right postcentral gyrus**	**Right supramarginal gyrus—superior division**	**EDI Subscale Total**	− **0.759**	**16**	**0.001**	**0.031**
Left central sulcus	Right supramarginal gyrus—posterior division	EDI Subscale Total	− 0.624	16	0.010	0.201
Left central sulcus	Left lateral occipital cortex—superior division	EDI Subscale Total	− 0.568	16	0.022	0.201
Left superior part of the precentral sulcus	Left precentral gyrus	EDI Subscale Total	− 0.580	16	0.018	0.201
Right subcentral gyrus	Left cingulate gyrus—posterior division	EDI Subscale Total	− 0.579	16	0.019	0.201
Right subcentral gyrus	Right angular gyrus	EDI Subscale Total	− 0.521	16	0.039	0.206
Right superior frontal gyrus	Right precentral gyrus	EDI Subscale Total	− 0.542	16	0.030	0.201
Left posterior ramus or segment of the lateral sulcus or fissure	Right angular gyrus	EDI Subscale Total	− 0.550	16	0.027	0.201
Right postcentral gyrus	Right supramarginal gyrus—superior division	Age of onset	− 0.574	16	0.020	0.321
Left central sulcus	Right supramarginal gyrus—posterior division	Age of onset	− 0.579	16	0.019	0.321
Right inferior part of the precentral sulcus	Right superior temporal gyrus—posterior division	Age of onset	0.549	16	0.028	0.333
Left posterior ramus or segment of the lateral sulcus or fissure	Right middle temporal gyrus—posterior division	Age of onset	− 0.666	16	0.005	0.235
Left hippocampus	Right supramarginal gyrus—posterior division	Time to treatment (months)	0.562	16	0.024	0.750
Right hippocampus	Left supramarginal gyrus—posterior division	Time to treatment (months)	0.539	16	0.031	0.750

Region	Clinical variable	*r*	*df*	*p*	*q*-value	
**Measure of centrality—strength**						
**Sensorimotor network**						
**Left central sulcus**	**EDI Body** Dissatisfaction	− **0.6322**	**16**	**0.009**	**0.021**	
Left paracentral lobule and sulcus	EDI Body Dissatisfaction	− 0.5239	16	0.037	0.053	
Left postcentral gyrus	EDI Body Dissatisfaction	− 0.5434	16	0.030	0.050	
**Left postcentral sulcus**	**EDI Body Dissatisfaction**	− **0.7065**	**16**	**0.002**	**0.019**	
Left postcentral sulcus	EDI Drive for Thinness	− 0.6648	16	0.005	0.061	
**Left postcentral sulcus**	**EDI Subscale Total**	− **0.6942**	**16**	**0.003**	**0.043**	
**Left precentral gyrus**	**EDI Body Dissatisfaction**	− **0.6873**	**16**	**0.003**	**0.019**	
Left precentral gyrus	EDI Drive for Thinness	− 0.5628	16	0.023	0.092	
Left precentral gyrus	EDI Subscale Total	− 0.5168	16	0.040	0.131	
Left subcentral gyrus and sulcus	EDI Body Dissatisfaction	− 0.5422	16	0.030	0.050	
**Right central sulcus**	**EDI Body Dissatisfaction**	− **0.6494**	**16**	**0.006**	**0.021**	
**Right paracentral lobule and sulcus**	**EDI Body Dissatisfaction**	− **0.679**	**16**	**0.004**	**0.019**	
Right paracentral lobule and sulcus	EDI Drive for Thinness	− 0.6359	16	0.008	0.061	
Right paracentral lobule and sulcus	EDI Subscale Total	− 0.5201	16	0.039	0.131	
Right postcentral gyrus	EDI Body Dissatisfaction	− 0.5196	16	0.039	0.053	
**Right postcentral sulcus**	**EDI Body Dissatisfaction**	− **0.6304**	**16**	**0.009**	**0.021**	
Right postcentral sulcus	EDI Drive for Thinness	− 0.5061	16	0.045	0.092	
Right postcentral sulcus	EDI Subscale Total	− 0.513	16	0.042	0.131	
**Right precentral gyrus**	**EDI Body Dissatisfaction**	− **0.6254**	**16**	**0.010**	**0.021**	

Bolded results represent *q*-signficant correlations

*r* Pearson’s correlation, *df* degrees of freedom, *p p*-value, *q* q-value

#### Measures of centrality

Regions within the sensorimotor network showing significant differences between AN and HC in *strength* were negatively correlated with the EDI Body Dissatisfaction scale, and the Subscale Total Scores. See Table 4.

## Discussion

Anorexia nervosa is a disorder characterized by a seeming lack of synchrony between physical needs and engagement in adaptive behaviors to address those needs (e.g., sensing and responding to hunger, seeming imperviousness to the discomfort of excessive exercise). Our findings showed that AN participants had lower connectivity and centrality within the sensorimotor network, which was associated with more negative evaluations of one’s body image as indexed by the Body Dissatisfaction subscale. We also found that AN participants had greater connectivity and centrality of the caudate nucleus. These findings suggest that individuals suffering from AN have reduced sensory propagation, input that may help guide adaptive behavior, and greater activation of the caudate nucleus, a region which has been shown to be involved in strategic planning, reward, and motor activity among other functions.

Prior work has examined the integrity of the somatosensory/sensorimotor networks and the basal ganglia network as neural substrates of these sensory experiences and rigid driven behaviors respectively. In this study, we aimed to further characterize the integrity of these networks by examining metric dynamics such as centrality within the somatosensory and basal ganglia networks. King et al. [43] described AN as a model of neuroplasticity in which the impact of potentially dangerous weight loss behaviors and the timing and duration of those behaviors could inform how the brain adapts to insult. As such, in this paper we examined not only group-level differences between those with a history of an AN diagnosis relative to typically developing controls, but also more continuous aspects of the disorder such as age of onset, time to treatment, and duration of the disorder. Our findings centered on two networks: the sensorimotor network and the basal ganglia network. Each will be discussed in turn.

First, we found pervasive weakened associations within areas of the sensorimotor network in adolescents with AN relative to HCs and weakened associations of the sensorimotor network with several other networks (i.e., occipital, default mode, central executive, emotion regulation), with the exception of the basal ganglia network in which there was increased connectivity. AN has been described as a disorder in which individuals are seemingly disconnected from somatic experiences: having difficulty labeling emotions, regulating emotions, responding adaptively to biological needs such as hunger and fatigue, and having an incoherent sense of identity often entangling their disorder with their experience of individuality and agency [64]. Thus, broadly, findings with the sensorimotor network are consistent with the phenomenology of AN: weakened associations between networks (sensorimotor) associated with the perception of afferent signals from the viscera and related changes to networks required to integrate such sensations to achieve adaptive actions and self-awareness.

Further, weakened connectivity and centrality within the sensorimotor cortex was associated with several clinical parameters. Greater subjective ratings on the EDI Body Dissatisfaction Subscale, and a combined total EDI subscale score were associated with weakened connectivity within the upper sensorimotor network. As these scales reflect beliefs and behaviors that are incongruent with somatic needs and drives (e.g., the Body Dissatisfaction Subscale assessing discontentment with the overall shape and size of body regions), the negative correlation of this scale with weakened connectivity of this network reflects worsening sensorimotor integration with elevated body dissatisfaction. While duration of illness was not significantly associated with connectivity within the sensorimotor network, a later age of onset was associated with weakened connectivity between regions of the sensorimotor networks and default mode network before correction for multiple comparisons. Past research has also shown similar results where greater sensorimotor network activity was associated with lower body dissatisfaction in control women [65]. At the network level, weakened centrality was documented in AN. Thus, our findings are consistent with prior work documenting weakened connectivity of thalamic and posterior insular regions suggesting weaker propagation of somatic signals to guide adaptive behaviors [29].

In contrast, stronger connectivity between the sensorimotor and basal ganglia networks may reflect the salience or rewarding value of body-related information, given the preoccupation with the body in AN. While somatic information and actions are not adaptively integrated in those with AN (e.g., hunger may not motivate eating), yet body-related information is rewarding in that it may reinforce maladaptive behavior. Thus, the connectivity of the sensorimotor and basal ganglia networks may reflect the salience of somatic signaling with action: albeit maladaptive. It is also important to note that in AN, activity of the caudate nucleus has been shown to be involved in strategic planning and consideration of consequences, as opposed to proximal hedonic responses [66]. Previous imaging studies on AN indicate greater caudate volumes [67] compared to controls, and greater glucose metabolism in the caudate compared to individuals with bulimia [68]. We also know that individuals with AN show greater functional activation in the caudate in response to monetary reward tasks [69]. Thus, findings of greater connectivity between the caudate and somatosensory cortex may indicate strategic planning; that is, alteration of behaviors towards long-term goals (i.e. avoiding food and getting thin) instead of short-term goal (i.e. eating food). Conversely, the basal ganglia network demonstrated weaker connectivity with the default mode network, perhaps reflecting that while focus on the somatic body may be negatively or positively reinforcing, focus on the self, as encapsulated by the self-awareness of the default mode, is not. Connectivity between these networks has been shown to play a key role in reward-based associative learning [70]. This may reflect that individuals with AN have difficulty differentiating experiences that will be rewarding [70].

### Limitations

Future studies should also investigate changes in cortico-striatal circuits longitudinally to understand the development of AN, as this cross-sectional study does not allow to make conclusions about causality. While the sample reflected individuals presenting to a specialized outpatient program for eating disorders, it was a mixed sample in the sense that individuals were at various phases of disorder and weight restoration. While such categorical distinctions of disorder, partial recovery, etc. are contested, nonetheless, defining categorical groups may have strengthened our interpretations. These limitations were addressed by examining more continuous measures of AN such as duration and time to treatment. Furthermore, while study participants were on stable doses of psychotrophic medications, a significant proportion of the clinical sample was on various medications and thus these medications may have influenced study results. Medication naïve samples offer a much stronger test and verification of study findings. Moreover, it is important to note that the associations between connectivity/graph theory measures and clinical measures are correlational and does not allow for casual inference. Finally, it should be noted that the atlas for parcellation of nodes was chosen based on prior research in anorexia nervosa. As there is no standard atlas to use for parcellating the brain, future research should test the effect of different brain atlases, as the selection of different atlases may impact final results [71, 72]. We used a structural atlas due to previous research conducted in patients with anorexia. The disadvantage of using a structural atlas is that it may average dissimilar functional signals based on an a-priori definition of a region.

## Conclusions and clinical implications

In the presence of a chronic medical condition, adolescents have been reported to develop a dislike and distrust of their bodies, feeling that their bodies had somehow let them down [73]. In AN, there may be a similar adversarial relationship with one’s body and the reported need to control one’s body to feel in control of oneself. Findings from the current study revealed weakened connectivity and centrality in the sensorimotor network, evidence suggesting that the strength, integrity, or efficiency in the processing of sensorimotor signals may be impaired. In adolescents with AN, the altered network metrics were associated with increased body dissatisfaction suggesting that perhaps unreliability of somatic signals can lead to negative evaluations of the body. The question whether such compromised network metrics are a consequence of repeatedly ignoring or being nonresponsive to bodily signals such as hunger or fatigue or whether such network metrics reflect a vulnerability to develop AN cannot be addressed in this cross-sectional study. To answer the question if the unreliability of the body necessitates the need for rigid rules and behaviors because one can’t “trust one’s body” is a topic needing further research. Regardless, our findings speak to the importance of intervention strategies that help individuals with AN to better sense, decipher, and act on the various interoceptive messages communicated by the body.

## Supplementary Information

**Additional file 1: Table 1**. Psychotropic Medications of Clinical Sample. Notes: 1. n = 12 individuals on medication, 3 on multiple medications. 2. For all short-acting and PRN medications (e.g., Lorazepam, Methylphenidate), participants were instructed not to take the medication the day of the scan. Compliance was assessed prior to scanning. There was no need to reschedule a scanning session based on this instruction. 12 individuals were on medication on the day of scanning; **Table 2**. Regions of Interest (ROIs). Sensorimotor Network: Thalamus [Includes Anterior, Central-medial Thalamus (Tha)], Hippocampus (Hip), Paracentral lobule and sulcus (PaCL/S), Primary Somatosensory Cortex/S1 [Includes Postcentral gyrus (PosCG), Postcentral sulcus (PosCS)], Central sulcus (Rolando's Fissure, CS), Precentral (Primary Motor Cortex/M1) [Includes Inferior part of the precentral sulcus (InfPrCS), Superior part of the precentral sulcus (SupPrCs), Precentral gyrus (PRCG)], Precuneus (PrCun), Secondary Somatosensory Cortex/S2 [Includes Subcentral gyrus (central operculum) and sulci (SbCG_S)], Supplementary Motor Area/M2 [Includes BA6/Superior Frontal Gyrus (SupFG), BA6/Superior Frontal Sulcus (SupFS), Posterior Insula (pINS) [Includes Long insular gyrus and central sulcus of the insula (LoInG/CInS), Inferior segment of the circular sulcus of the insula (InfCirInS), Posterior ramus (or segment) of the lateral sulcus (or fissure) (PosLS)]. Basal Ganglia Network: Putamen (Pu), Caudate nucleus (CaN), Nucleus Accumbens (Nacc)], Globus Pallidus [Includes Pallidum (Pal)], Brain Stem (Bstem).** Figure 1** Regions of Interest (ROIs): Tha: thalamus, Hipp: hippocampus, PaCL: paracentral lobule, PosCG: postcentral gyrus, PosCS: postcentral sulcus, CS: central sulcus, InfPrCS; inferior part of the precentral sulcus, SupPrCS; superior part of the precentral sulcus, PrCG: precentral gyrus, PrCu: precuneus, SubCG/S: subcentral gyrus and sulcus, SupFG: superior frontal gyrus, SupFS: superior frontal sulcus, SupCirIns: superior part of the circular sulcus of the insula, InfCirIns: inferior part of the circular sulcus of the insula, LoInG/CInS: long insular gyrus and central sulcus of the insula, PosLS: posterior ramus of the lateral sulcus, Put: putamen, CaN: caudate nucleus, NAcc: nucleus accumbens, Pal: pallidum, BStem: brainstem.

## Data Availability

All data and methods used in the current manuscript are freely available from the authors upon reasonable request.

## Abbreviations

AN: Anorexia nervosa
HC: Healthy control
EDI: Eating disorders inventory
MRI: Magnetic resonance imaging
fMRI: Functional magnetic resonance imaging
ROI: Region of interest
BOLD: Blood oxygen level dependent
GLM: General linear model
FDR: False discovery rate
