# Supplementary material for: Complex functional brain network properties in anorexia nervosa
Source: J Eat Disord. 2022 Feb 5;10:13. doi: 10.1186/s40337-022-00534-9 (PMC8817538; doi:10.1186/s40337-022-00534-9)
Supplement: Supplementary file 1 — Additional file 1: Table 1. Psychotropic Medications of Clinical Sample. Notes: 1. n = 12 individuals on medication, 3 on multiple medications. 2. For all short-acting and PRN medications (e.g., Lorazepam, Methylphenidate), participants were instructed not to take the medication the day of the scan. Compliance was assessed prior to scanning. There was no need to reschedule a scanning session based on this instruction. 12 individuals were on medication on the day of scanning; Table 2. Regions of Interest (ROIs). Sensorimotor Network: Thalamus [Includes Anterior, Central-medial Thalamus (Tha)], Hippocampus (Hip), Paracentral lobule and sulcus (PaCL/S), Primary Somatosensory Cortex/S1 [Includes Postcentral gyrus (PosCG), Postcentral sulcus (PosCS)], Central sulcus (Rolando's Fissure, CS), Precentral (Primary Motor Cortex/M1) [Includes Inferior part of the precentral sulcus (InfPrCS), Superior part of the precentral sulcus (SupPrCs), Precentral gyrus (PRCG)], Precuneus (PrCun), Secondary Somatosensory Cortex/S2 [Includes Subcentral gyrus (central operculum) and sulci (SbCG_S)], Supplementary Motor Area/M2 [Includes BA6/Superior Frontal Gyrus (SupFG), BA6/Superior Frontal Sulcus (SupFS), Posterior Insula (pINS) [Includes Long insular gyrus and central sulcus of the insula (LoInG/CInS), Inferior segment of the circular sulcus of the insula (InfCirInS), Posterior ramus (or segment) of the lateral sulcus (or fissure) (PosLS)]. Basal Ganglia Network: Putamen (Pu), Caudate nucleus (CaN), Nucleus Accumbens (Nacc)], Globus Pallidus [Includes Pallidum (Pal)], Brain Stem (Bstem). Figure 1 Regions of Interest (ROIs): Tha: thalamus, Hipp: hippocampus, PaCL: paracentral lobule, PosCG: postcentral gyrus, PosCS: postcentral sulcus, CS: central sulcus, InfPrCS; inferior part of the precentral sulcus, SupPrCS; superior part of the precentral sulcus, PrCG: precentral gyrus, PrCu: precuneus, SubCG/S: subcentral gyrus and sulcus, SupFG: superior frontal gyrus, SupFS: superior frontal sulcu [file 40337_2022_534_MOESM1_ESM.docx]

| **Supplemental Table 1. Psychotropic Medications of Clinical Sample^1^** | |
| --- | --- |
| **Pharmacological Class**  **Compound Name** | **Number of Individuals** |
| Selective Serotonin Reuptake Inhibitors |  |
| Fluoxetine | 5 |
| Citalopram | 2 |
| **Escitalopram** | 2 |
| Sertraline | 1 |
| Selective Serotonin Reuptake Inhibitors and  Sigma-1 Receptor Agonist |  |
| Fluvoxamine | 1 |
|  |  |
| Atypical Antipsychotics |  |
| Quetiapine | 1 |
| Benzodiazepine |  |
| Lorazepam^2^ | 1 |
| Aminoketone |  |
| Buproprion | 1 |
| Stimulant |  |
| Methylphenidate^2^ | 1 |

Notes: 1. n=12 individuals on medication, 3 on multiple medications. 2. For all short-acting and PRN medications (e.g, Lorazepam, Methylphenidate), participants were instructed not to take the medication the day of the scan. Compliance was assessed prior to scanning. There was no need to reschedule a scanning session based on this instruction. 12 individuals were on medication on the day of scanning.

**Supplemental Table 2. Regions of Interest (ROIs)**

Sensorimotor Network: Thalamus [Includes Anterior, Central-medial Thalamus (Tha)], Hippocampus (Hip), Paracentral lobule and sulcus (PaCL/S), Primary Somatosensory Cortex/S1 [Includes Postcentral gyrus (PosCG), Postcentral sulcus (PosCS)], Central sulcus (Rolando's Fissure, CS), Precentral (Primary Motor Cortex/M1) [Includes Inferior part of the precentral sulcus (InfPrCS), Superior part of the precentral sulcus (SupPrCs), Precentral gyrus (PRCG)], Precuneus (PrCun), Secondary Somatosensory Cortex/S2 [Includes Subcentral gyrus (central operculum) and sulci (SbCG_S)], Supplementary Motor Area/M2 [Includes BA6/Superior Frontal Gyrus (SupFG), BA6/Superior Frontal Sulcus (SupFS), Posterior Insula (pINS) [Includes Long insular gyrus and central sulcus of the insula (LoInG/CInS), Inferior segment of the circular sulcus of the insula (InfCirInS), Posterior ramus (or segment) of the lateral sulcus (or fissure) (PosLS)]

Basal Ganglia Network: Putamen (Pu), Caudate nucleus (CaN), Nucleus Accumbens (Nacc)], Globus Pallidus [Includes Pallidum (Pal)],

Brain Stem: Brain Stem (Bstem).

|  | **Region** | **Full Destrieux Name** | **Destrieux Label** |  |
| --- | --- | --- | --- | --- |
| **Somatosensory Network** | | | |  |
|  |  |  |  |  |
| 1 | Thalamus | Thalamus (Anterior, Central-medial) | Tha |  |
| 2 | Hippocampus | Hippocampus | Hipp |  |
| 3 | Paracentral lobule and sulcus | Paracentral lobule and sulcus | PaCL/S |  |
| 4 | Primary Somatosensory Cortex (Postcentral Gyrus/S1) | Postcentral gyrus | PosCG |  |
| 5 |  | Postcentral sulcus | PosCS |  |
| 6 | Central Sulcus (Primary Somatosensory Cortex/S1) | Central sulcus (Rolando's Fissure) | CS |  |
| 7 |  | Inferior part of the precentral sulcus | InfPrCS |  |
| 8 | Precentral (Primary Motor Cortex, M1) | Superior part of the precentral sulcus | SupPrCs |  |
| 9 |  | Precentral gyrus | PRCG |  |
| 10 | Precuneus | Precuneus | PrCun |  |
| 11 | Secondary Somatosensory Cortex (S2) | Subcentral gyrus (central operculum) and sulci | SbCG/S |  |
| 12 | Supplementary Motor Area (SMA/M2) | BA6/Superior Frontal Gyrus | SupFG |  |
| 13 |  | BA6/Superior Frontal Sulcus | SupFS |  |
| 14 | Middle Insula | Superior segment of the circular sulcus of the insula | SupCirInS |  |
| 15 |  | Long insular gyrus and central sulcus of the insula | LoInG/CInS |  |
| 16 | Posterior Insula | Inferior segment of the circular sulcus of the insula | InfCirInS |  |
| 17 |  | Posterior ramus (or segment) of the lateral sulcus (or fissure) | PosLS |  |
| **Basal Ganglia** | | | |  |
| 1 |  | Putamen | Pu |  |
| 2 | Basal Ganglia | Caudate nucleus | CaN |  |
| 3 |  | Nucleus Accumbens | Nacc |  |
| 4 | Globus Pallidus | Pallidum | Pal |  |
| **Brainstem** | | | |  |
| 5 | Periaqueductal Gray (PAG) | Brainstem | Bstem |  |


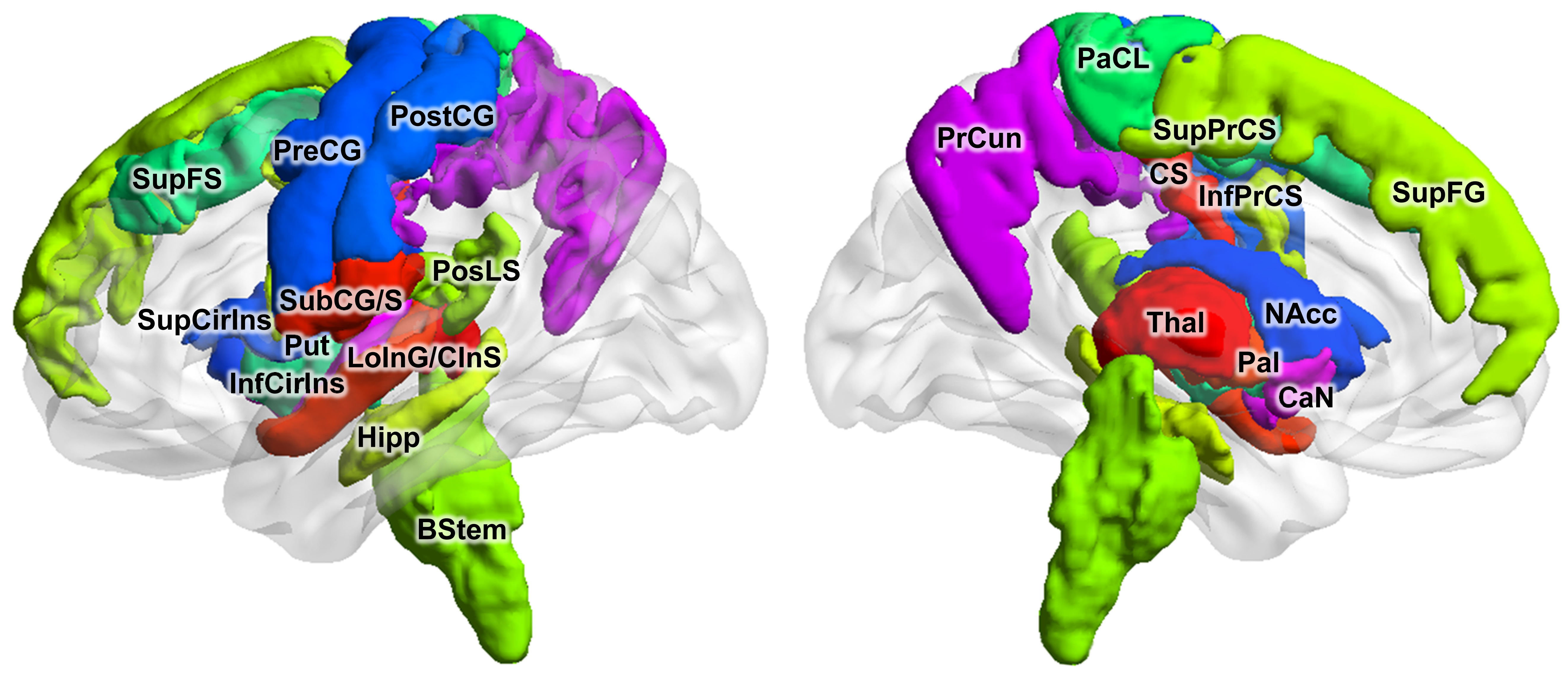


**Supplemental Figure 1 – Regions of Interest (ROIs):** Tha: thalamus, Hipp: hippocampus, PaCL: paracentral lobule, PosCG: postcentral gyrus, PosCS: postcentral sulcus, CS: central sulcus, InfPrCS; inferior part of the precentral sulcus, SupPrCS; superior part of the precentral sulcus, PrCG: precentral gyrus, PrCu: precuneus, SubCG/S: subcentral gyrus and sulcus, SupFG: superior frontal gyrus, SupFS: superior frontal sulcus, SupCirIns: superior part of the circular sulcus of the insula, InfCirIns: inferior part of the circular sulcus of the insula, LoInG/CInS: long insular gyrus and central sulcus of the insula, PosLS: posterior ramus of the lateral sulcus, Put: putamen, CaN: caudate nucleus, NAcc: nucleus accumbens, Pal: pallidum, BStem: brainstem
